# Improving the implementation of perioperative safety guidelines using a multifaceted intervention approach: protocol of the IMPROVE study, a stepped wedge cluster randomized trial

**DOI:** 10.1186/s13012-014-0198-5

**Published:** 2015-01-08

**Authors:** Yvette EJJM Emond, Hiske Calsbeek, Steven Teerenstra, Gerrit JA Bloo, Gert P Westert, Johan Damen, André P Wolff, Hub C Wollersheim

**Affiliations:** Radboud university medical center, Radboud Institute for Health Sciences, IQ healthcare, PO Box 9101, 114 IQ healthcare, 6500 HB Nijmegen, The Netherlands; Radboud university medical center, Radboud Institute for Health Sciences, Department of Anesthesiology, Pain and Palliative Care, Nijmegen, The Netherlands; Radboud university medical center, Radboud Institute for Health Sciences, Dapartment for Health Evidence, section Biostatics, Nijmegen, The Netherlands

**Keywords:** Guideline adherence, Health-care quality indicators, Implementation, Multifaceted approach, Patient safety, Perioperative care, Stepped wedge design

## Abstract

**Background:**

This study is initiated to evaluate the effects, costs, and feasibility at the hospital and patient level of an evidence-based strategy to improve the use of Dutch perioperative safety guidelines. Based on current knowledge, expert opinions and expertise of the project team, a multifaceted implementation strategy has been developed.

**Methods/design:**

This is a stepped wedge cluster randomized trial including nine representative hospitals across The Netherlands. Hospitals are stratified into three groups according to hospital type and geographical location and randomized in terms of the period for receipt of the intervention. All adult surgical patients meeting the inclusion criteria are assessed for patient outcomes. The implementation strategy includes education, audit and feedback, organizational interventions (e.g., local embedding of the guidelines), team-directed interventions (e.g., multi-professional team training), reminders, as well as patient-mediated interventions (e.g., patient safety cards). To tailor the implementation activities, we developed a questionnaire to identify barriers for effective guideline adherence, based on (a) a theoretical framework for classifying barriers and facilitators, (b) an instrument for measuring determinants of innovations, and (c) 19 semi-structured interviews with perioperative key professionals. Primary outcome is guideline adherence measured at the hospital (i.e., cluster) and patient levels by a set of perioperative Patient Safety Indicators (PSIs), which was developed parallel to the perioperative guidelines. Secondary outcomes at the patient level are in-hospital complications, postoperative wound infections and mortality, length of hospital stay, and unscheduled transfer to the intensive care unit, non-elective readmission to the hospital and unplanned reoperation, all within 30 days after the initial surgery. Also, patient safety culture and team climate will be studied as potential determinants. Finally, a process evaluation is conducted to identify the compliance with the implementation strategy, as well as an economic evaluation to assess the costs. Data sources are registered clinical data and surveys. There is no form of blinding.

**Discussion:**

The perioperative setting is an unexplored area with respect to implementation issues. This study is expected to yield important new evidence about the effects of a multifaceted approach on guideline adherence in the perioperative care setting.

**Trial registration:**

Dutch trial registry: NTR3568

**Electronic supplementary material:**

The online version of this article (doi:10.1186/s13012-014-0198-5) contains supplementary material, which is available to authorized users.

## Background

The focus of this project is to implement perioperative safety guidelines in Dutch hospitals. Perioperative adverse events (AEs) are associated with deaths, injured and disabled patients, and high costs [1-5]. In the Netherlands, about 1.2 million surgeries are performed annually [6]. AEs occur in 7.1% of all hospital patients [7]. Two thirds (65%) of all hospital AEs are associated with perioperative care [8]. Research shows that perioperative mortality ranges between 1.5% and 3.13% [9-12], while 47%–62% is considered avoidable [2,8]. Analyses by the Dutch Health Care Inspectorate (IGZ) showed that perioperative care in the Netherlands was lacking standards in information transfer, clinical documentation, teamwork, and coordination [13-15]. In response to the recommendations of the IGZ, national evidence-based perioperative safety guidelines including Patient Safety Indicators (PSIs) were developed (2010–2013) [16-18].

Implementation of these guidelines is expected to reduce adverse patient outcomes. In addition, several studies showed that using stop moments and the timely administration of antibiotic prophylaxis improve perioperative outcome [3,9,12]. Adoption of guidelines in clinical practice has, however, proven to be difficult. About 30%–40% of all patients do not receive care according to actual scientific knowledge [19]. Many approaches claim to offer solutions to this problem, but many aspects of the implementation process are also still unknown. From the extensive review of Grimshaw et al. [20], we know that some implementation activities are in potential very successful. However, the extent of success varies between settings and topics, and knowledge about implementation activities for guidelines or protocols regarding perioperative care is lacking. It is therefore important to derive a potentially successful strategy and study its effect, costs, and feasibility in current practice. It is, however, impossible to deliver such a strategy simultaneously to all hospitals because of logistical, practical, and financial reasons. For that reason, a stepped wedge cluster randomized trial design is chosen. This design is also considered advantageous when there is a belief that the implementation strategy will do more good than harm (making a parallel design, in which certain hospitals do not receive the intervention or to withdraw the intervention as would occur in a cross-over design, is unethical) and it furthermore minimizes contamination [21].

## Objective

The aim of this study is to investigate the (cost) effectiveness of a multifaceted approach to implement the perioperative safety guidelines. Our main research question is

1. Can we improve the use of the national perioperative safety guidelines at the hospital and patient levels? In other words, does a multifaceted approach to implement the perioperative guidelines lead to a higher compliance than unsupported implementation?
  Other research questions are the following:
2. Does the perioperative patient safety (i.e., patient outcomes) improve with better adherence to the guidelines?
3. What are the costs of this implementation strategy and what is the cost-effectiveness at the hospital level?
4. What is the feasibility of this implementation strategy at the hospital level?

In this study protocol, we describe a stepped wedge cluster randomized trial design to evaluate the effect of a multifaceted implementation strategy on guideline adherence and patient safety in Dutch hospitals (research questions 1 and 2). We also describe the economic evaluation (research question 3) and process evaluation (research question 4).

## Methods/design

### Study design

Implementation of Perioperative Safety Guidelines (in Dutch: Implementatie Richtlijnen Operatieve Veiligheid (IMPROVE)) is a multicenter study in nine hospitals using an one-way (unidirectional) cross-over cluster trial design (i.e., the clusters cross over in one direction only, from control to intervention) where each participating cluster receives both intervention and control treatments consecutively, in separate periods.

A stepped wedge design is a sequential roll-out of an intervention (here: the multifaceted implementation strategy) to participants (individuals or clusters of individuals, here: groups of hospitals) over a number of time periods. All groups (hence all hospitals) start with the control situation (no IMPROVE implementation activities) at the beginning of the study. At each time point, a new group of three hospitals crosses over from the control situation to the implementation situation. Good balance of hospital characteristics (e.g., hospital type and geographical location) over the groups (of three hospitals) was achieved by matching. Each group will start the implementation phase of 4 months at a different time point, directly after one of the measurements (T0, T1, T2). The time point a group crosses over is randomized (over the groups). Randomization was computer generated by an independent statistician when recruitment of clusters was complete (Additional file 1). By the end of the study, all groups (hence all hospitals) will have received the IMPROVE implementation strategy. The intervention is never removed once it has been implemented, at least over the course of the trial.

In our protocol, hospitals cross over from control to implementation period at three time points (Table 1). A stepped wedge design incorporates data collection in all hospitals at each time point [21,22]. The first time point corresponds to a baseline measurement where none of the hospitals have received the intervention of interest (i.e., multifaceted implementation approach). After the baseline measurement, the first group of hospitals receives the implementation strategy. After the second measurement, the next group of hospitals receives the implementation strategy, and at the third time point, the final group switches from usual care to the implementation phase, so that finally, all included hospitals have received the implementation activities. In this way, comparisons within hospitals and between hospitals will be available, making the design powerful.

**Table 1:** **Stepped wedge design with four steps in which ‘C’ represents the control situation and ‘I’ represents the intervention phase**

**Group**	**Measurement**			
	**T0**	**T1**	**T2**	**T3**
**1**	C	I	I	I
**2**	C	C	I	I
**3**	C	C	C	I

In this study, nine hospitals are assigned into three groups of three hospitals. Data collection runs parallel for each hospital. At baseline measurement (T0), all primary and secondary outcomes will be measured per patient (*N* = 50) per hospital. In the interval between T0 and T1, implementation activities will take place in the first group of three hospitals. After the second measurement (T1), the second group of three hospitals crosses over to the intervention phase, while implementation activities continue in the first group and so on. At the final measurement (T3), all hospitals will be in the intervention phase (see Table 1).

### Study population

This study comprises nine hospitals in the Netherlands: two academic, four tertiary teaching, and three regional hospitals, with 200 to up to more than 1,300 beds each. Eight hospitals have a NIAZ accreditation. This accreditation of the Dutch Institute for Health Care Accreditation (NIAZ, part of the International Society for Quality in Healthcare) is provided to hospitals that meet international standards developed and tested for external evaluation of health-care organizations [23]. Two hospitals are part of the Dutch Federation of University Medical Centers (NFU) and provide the most specialized care [24]. Four hospitals are part of the Association of Tertiary Medical Teaching Hospitals (STZ) and provide highly specialized medical care (the next level of specialization below that of NFU hospitals) [25]. Although the hospitals are not randomly chosen, the stratification in three classes is a strong point, and we believe these hospitals represent the practice of Dutch hospital care. Within these clusters, we assess 1,800 surgical patients distributed over four measurement points, i.e., 450 patients per measurement point with 50 patients per hospital (see the “Power calculation” section below). The study focuses on patients undergoing elective abdominal or vascular surgery with a mortality risk ≥1% [10]. These surgeries are selected because of the estimated higher risk of complications and hospital mortality, as the number of included surgical patients is otherwise too small to be able to find significant effects of guideline adherence on perioperative patient safety (second research question). Exclusion criteria are the following: 1) patients younger than 18 years, 2) patients in day care (1-day hospital stays are defined as hospital admissions of 24 h or less), 3) patients undergoing cardiac surgery, 4) patients undergoing organ transplantations (except kidney transplants), and 5) patients undergoing emergency surgery, i.e., surgery needed within 24 h and/or without an pre-anesthesia evaluation record available and/or surgery with a start time after 4.59 PM or before 7.30 AM or during the weekend. Key disciplines are also involved as study objects: surgeons, anesthesiologists, OR, anesthesia, recovery, and ward nurses, and ICU employees.

### The intervention

The intervention consists of a multifaceted implementation strategy directed at the cluster. Each group of hospitals will be exposed to the intervention for 4 months. The content of the multifaceted approach is based on 1) scientific literature (systematic review of interventions by Grimshaw et al. [20,26]); 2) expert opinion: prioritizing implementation activities by 24 clinical and implementation experts (RAND modified Delphi method); 3) knowledge and experience of the IMPROVE team regarding the feasibility of the interventions (estimated costs, effort, and time for the hospitals as well as the implementation team). This resulted in a standard package including five activities (Table 2): 1. small educational meetings, 2. audit and feedback (based on local indicator scores and benchmarking, results of surveys on team climate and safety culture, and barriers), 3. structured observation rounds with feedback, 4. integration of the guidelines in local activities and processes, and 5. the use of patient safety cards. A set of six additional activities are offered optional: 1. personal information letter in the mailbox, 2. exchange platform for sharing information between hospitals within a group, 3. scan of the total perioperative process, 4. posters showing details of the perioperative care process, 5. reminder flashes via e-mail, and 6. team training.

**Table 2:** **Description of the content of the implementation activities in the IMPROVE standard and additional packages**

**Implementation activities**	**Description**
**Standard package**	
Small-scale educational meetings	Workshop or skills training for perioperative key disciplines including assignments, role playing, own presentations, patient stories or discussion, and problem solving of hypothetical patient situations/case studies. Provided by an opinion leader within the field of patient safety or a highly respected colleague. Based on active participation in small groups: multi- or mono-disciplinary groups (i.e., per discipline, e.g., surgeons and recovery nurses separately). The content is based on the key constraints and the most important obstacles in applying the guidelines for a hospital (based on the results of the audit) and a brainstorming session during the training or pre-handed topics participants find important to discuss.
Audit and feedback	Feedback is based on the indicator measurement(s), structural observation, barrier analysis, and the TCI and HSOPS questionnaires. The feedback consists of a local paper report with the hospital’s own results, benchmarked, and presented in relation to all nine participating hospitals. The hospitals in the intervention phase receive this report shortly after a measurement period. The feedback report is presented and discussed with the key professionals in a meeting.
Structural observation	Observation by a trained expert of the pre-, per-, and postoperative trajectory of one surgical patient (on the ward, operation room, and recovery ward) based on a structured observation list. Feedback is based on the completed observation list. The hospitals receive the feedback immediately afterwards. Also, the structured observation list used is then made available to the hospital. In this way, the hospital is able to perform its own observations of the perioperative process.
Local embedding of the guidelines	Concrete and visible integration into and/or completion of a local protocol and/or checklist, for example, the adaptation of the guidelines in a local protocol; conducting audits (indicator measurements), structural observations, and visitation to monitor the implementation of the guidelines; the use of reminder systems (completing existing checklists based on the guidelines, if possible, new digital checklist may be installed in electronic patient records); decision support and feedback on the implementation of the protocol (using ICT); and incorporation of the guidelines in the clinical pathway, e.g., resignation letter to the general practitioner.
Patient safety cards	Two patient safety cards (each with six cartoons on the front and explanations on the back) based on the perioperative guidelines, entitled “Help us with your safe surgery” and “Discharge from the hospital” are sent to the patients with an accompanying letter in preparation of the preoperative screening or admission to the hospital.
	Both patient safety cards are offered again to the patients on the preoperative outpatient clinic and on the nursing ward, respectively.
	Medical specialist and nurses discuss the patient safety cards with the patient in order to explicitly invite patients to ask questions and to attend caregivers on parts of the cards during their health-care process.
**Additional package**	
Personal information letter in the mailbox	Personal information letter to all key disciplines about the (use of the) guidelines.
Exchange platform	This is a platform for the hospitals within a group to exchange their best practices, ideas, and experiences with implementing the guidelines.
Scan of the total perioperative process	A practice scan consisting of five parts:
	-Hospital staff complete an online questionnaire about the perioperative process (via e-mail with a login code)
	-Interviews with hospital staff for more background information (based on remarkable answers in the questionnaire)
	-Structured observation on side
	-Paper report by post (the report contains the findings, a top five of strengths and weaknesses, and recommendations)
	-Feedback meeting to discuss the report
Electronic reminder message	Catchy quote on behalf of an opinion leader within the perioperative process of a hospital. The content is based on the audit results; a recommendation comes in the spotlight which proves to be a bottleneck for the hospital.
Posters	Visual representation of e.g., the stop moments and the perioperative trajectory of the patient, shown as a subway line.
Multi-professional team training	The IMPROVE team facilitates contacts between the participating hospitals and organizations which provide trainings aimed at improving team culture, like crew resource management.

The activities are tailor-made, i.e., the content depends on the analysis of the current performance and guideline adherence (indicator measurement, structured observation of the perioperative care process, and the surveys on team climate and patient safety culture), local barriers in the perioperative setting (barrier analysis), local wishes, and initiatives already realized in the hospital.

### Barrier analysis

We developed a questionnaire to identify barriers for effective guideline adherence experienced by the mentioned professions, as insight into the barriers and facilitators of guideline adherence is essential in deciding what kinds of implementation activities should be developed. This questionnaire is based on (a) the theoretical framework for classifying barriers and facilitators as described by Van Sluisveld et al. [27], (b) the instrument for measuring determinants of innovations by the Dutch Organization for Applied Scientific Research [28], (c) 19 individual semi-structured interviews by the developers of the perioperative safety guidelines. The questionnaire contains questions about demographic characteristics and statements concerning barriers and facilitators regarding perioperative guideline adherence. Professionals are also asked to prioritize their barriers into a personal top three list. Barriers and facilitators are then analyzed and grouped into the context of the intervention characteristics, the societal context, the implementation characteristics, the institutional characteristics, the social context, the professional characteristics, and the patient characteristics.

### Outcome measures

Several outcomes are measured at the hospital (i.e., the cluster) and the patient levels (Table 3). The primary outcome measure is guideline adherence according to the PSIs as defined in the national indicator set. This set comprises nine indicators on the processes and structures of care [18]. Secondary (patient) outcomes are in-hospital complications (with particular attention to postoperative wound infections) and hospital mortality, as well as length of hospital stay (LoHS), unscheduled transfer to the intensive care unit (ICU), non-elective hospital readmission, and unplanned reoperation, which are derived from a maximum period of 30 days after the initial surgery.

**Table 3:** **Methods and instruments for measuring the effects of a multifaceted implementation strategy to implement the Dutch national perioperative safety guidelines**

**Outcome variable**	**Data source (type of measurement)**	**Frequency of measurement and sample size per measurement**	**Moment of measurement**	**Unit of analysis**
**Primary outcome** (guideline adherence measured by an indicator set of nine process and structure indicators)				
Completion of the total STOP bundle (seven separate stop moments in the perioperative care process), % patients	Retrospective patient record review in the hospital information systems	4 (T0 (before), T1, T2, T3 (after) measurement) *N* = 1,800	−3 months, +4 months, +11 months and +18 months	Patient
Availability of a protocol on antibiotic use	Short questionnaire on the underlying items of the structure indicators	4 (T0 (before), T1, T2, T3 (after) measurement) *N* = 1	−3 months, +4 months, +11 months and +18 months	Hospital
Timely administration of antibiotic prophylaxis, % patients	Retrospective patient record review in the hospital information systems	4 (T0 (before), T1, T2, T3 (after) measurement) *N* = 1,800	−3 months, +4 months, +11 months and +18 months	Patient
Availability of a protocol on anticoagulant use	Short questionnaire on the underlying items of the structure indicators	4 (T0 (before), T1, T2, T3 (after) measurement) *N* = 1	−3 months, +4 months, +11 months and +18 months	Hospital
Availability of a protocol on responsibilities regarding maintenance of medical equipment	Short questionnaire on the underlying items of the structure indicators	4 (T0 (before), T1, T2, T3 (after) measurement) *N* = 1	−3 months, +4 months, +11 months and +18 months	Hospital
Availability of a protocol on (performing) prospective risk analysis of medical equipment	Short questionnaire on the underlying items of the structure indicators	4 (T0 (before), T1, T2, T3 (after) measurement) *N* = 1	−3 months, +4 months, +11 months and +18 months	Hospital
Availability of OR regulations	Short questionnaire on the underlying items of the structure indicators	4 (T0 (before), T1, T2, T3 (after) measurement) *N* = 1	−3 months, +4 months, +11 months and +18 months	Hospital
Presence of a surveillance system for postoperative wound infections	Short questionnaire on the underlying items of the structure indicators	4 (T0 (before), T1, T2, T3 (after) measurement) *N* = 1	−3 months, +4 months, +11 months and +18 months	Hospital
Presence of a morbidity and mortality registration	Short questionnaire on the underlying items of the structure indicators	4 (T0 (before), T1, T2, T3 (after) measurement) *N* = 1	−3 months, +4 months, +11 months and +18 months	Hospital
**Secondary outcomes**				
Complications (in-hospital), % patients	Retrospective patient record review in the hospital information systems	4 (T0 (before), T1, T2, T3 (after) measurement) *N* = 1,800	−3 months, +4 months, +11 months and +18 months	Patient
Postoperative wound infections (in-hospital), % patients	Retrospective patient record review in the hospital information systems	4 (T0 (before), T1, T2, T3 (after) measurement) *N* = 1,800	−3 months, +4 months, +11 months and +18 months	Patient
Postoperative mortality (in-hospital), % patients	Retrospective patient record review in the hospital information systems	4 (T0 (before), T1, T2, T3 (after) measurement) *N* = 1,800	−3 months, +4 months, +11 months and +18 months	Patient
Length of hospital stay, number of days	Retrospective patient record review in the hospital information systems	4 (T0 (before), T1, T2, T3 (after) measurement) *N* = 1,800	−3 months, +4 months, +11 months and +18 months	Patient
Unscheduled transfer to the ICU (within 30 days after the initial surgery), % patients	Retrospective patient record review in the hospital information systems	4 (T0 (before), T1, T2, T3 (after) measurement) *N* = 1,800	−3 months, +4 months, +11 months and +18 months	Patient
Non-elective hospital readmission (within 30 days after the initial surgery), % patients	Retrospective patient record review in the hospital information systems	4 (T0 (before), T1, T2, T3 (after) measurement) *N* = 1,800	−3 months, +4 months, +11 months and +18 months	Patient
Unscheduled reoperation (within 30 days after the initial surgery), % patients	Retrospective patient record review in the hospital information systems	4 (T0 (before), T1, T2, T3 (after) measurement) *N* = 1,800	−3 months, +4 months, +11 months and +18 months	Patient
**Determinants of change**				
Team climate	Team climate inventory	1 (T0 (before) measurement)	−3 months	Professional
Patients safety culture	Hospital survey on patient safety culture	1 (T0 (before) measurement)	−3 months	Professional
**Barriers and facilitators of guideline adherence**	Questionnaire	1 (before the start of the implantation period in the concerning group of hospitals) *N* ≥ 7 (at least one surgeon, anesthesiologist, OR, anesthesia, recovery, and ward nurse, and ICU employee)	Variable (depending on the start of the implementation period)	Professional

The rationale for these secondary outcomes is based on a prioritization procedure by 16 perioperative health-care professionals in the nine participating hospitals: they were invited to list ten outcomes with the highest clinical relevance to perioperative care.

#### Definitions of outcomes at patient level

We define a complication as an unintended and unwanted event or state during or following medical treatment that has an unfavorable effect on the health of the patient to such an extent that adjustment of the medical treatment is necessary or irreparable harm has occurred [29]. Postoperative wound infections are divided into superficial and deep wound infections, with one of the following three symptoms present: pain and or sensitivity, local swelling, redness and or warmth [30]. Postoperative mortality is the death of a patient as the result of any perioperative complication. Length of hospital stay is defined as the time period in days between admission and discharge. An unplanned reoperation is any secondary surgical procedure required as a result of a complication directly or indirectly related to the index operation [31]. A non-elective readmission is an unintended, acute readmission from the index admission [32]. A readmission includes at least one overnight stay. An unscheduled transfer to the ICU is identified when an admission to the ICU department has not been ordered preoperatively. All these data are obtained from the hospital information systems.

### Data sources

Information about the processes and outcomes of perioperative care are retrospectively derived from patient medical records in the participating hospitals by two researchers (YE and GB). The first 50 patients undergoing an initial elective surgery within the measurement period and who meet the inclusion criteria are selected from the hospital databases.

To measure the structure indicators of the national indicator set, the contact person in each hospital is invited to fill out a short questionnaire on the underlying items of the structure indicators on a dichotomous scale (yes/no).

Health-care providers in the concerning hospitals are invited to respond to two different questionnaires and additional background questions on gender, medical position, and years of experience in the current function and in the current hospital. The Team Climate Inventory (TCI) [33] and the Hospital Survey on Patient Safety Culture (HSOPS) [34] are used to analyze whether team climate and patient safety culture are determinants of guideline adherence. The TCI measures the climate of the various teams of health-care providers. Good team work is essential to provide proper and safe care [35-37]. The TCI is a valid, reliable, and discriminating self-report measure of the climate of a hospital team. The TCI is based on a four-factor theory of team climate for innovation and assesses the factors, vision, participative safety, task orientation, and support for innovation in 13 subscales [33]. To measure the patient safety culture, we use the HSOPS [34,38]. The HSOPS is a valid and reliable survey for assessing the patient safety culture in hospitals. It measures 12 dimensions of patient safety culture on the basis of ideas of unsafely designed care processes or systems that increase the likelihood of the occurrence of adverse events.

### Power calculation

The key characteristic of cluster randomized trials (CRTs) is that the individual units (here: patients) within a cluster (here: hospital) are correlated, and this feature must be incorporated into power calculations and trial analysis [22]. Besides the intracluster correlation (ICC), the power calculation should also take into account the number of steps and treatment delay as the power depends on the number of steps (or measurements) and decreases by a delay in intervention effect.

We made the following assumptions: 1) nine hospitals in the Netherlands (10% of the total number of hospitals) [39] will cooperate in this study; 2) an average effect of 10%–15% increase in the process indicators (primary outcome measure) might be expected from the literature [20]; 3) baseline adherence to the guidelines is unknown, but a 15% increase from a baseline level of 50% requires the largest sample size and therefore the sample size calculated will also suffice if the baseline adherence is different from 50%; and 4) ICC is unknown but can be estimated between 0.1 and 0.3 as ICCs have shown to vary between 0 and 0.4 with a median ICC of 0.06 in other cluster randomized trials in secondary care [40]. Also, using process variables results in a comparable range and median of ICCs [40]. Based on the availability of nine hospitals, a baseline adherence of 50%, an increase of 15% by the intervention, a significance level alpha of 0.05, an ICC between 0.1 and 0.3, and four time points (three steps in the stepped design), 50 patients per cluster (equal sized) and time point are needed to reach a power of at least 0.85, using the formula of Hussey and Hughes [22]. As a result, 1,800 (50 patients × 9 hospitals × 4 measurements) patients will be included over all hospitals in the course of the trial.

### Analysis

After each measurement period, data are manually checked to identify out-of-range answers, inconsistent or missing data. Because of the hierarchical design (patients nested within hospitals), multilevel linear and logistic regression analysis will be used to compare the outcomes between treatment groups, with age, sex, the American Society of Anesthesiologists (ASA) physical status classification, and socioeconomic status (SES) as covariates. The ASA physical status classification is a five-point scale ranging from ASA 1 (healthy patient) to ASA 5 (moribund patient not expected to survive without surgery, e.g., a patient with a ruptured aortic aneurysm) [41]. Time will be included as fixed effect [22]. The association between guideline adherence and patient outcomes will also be analyzed to determine whether better adherence leads to better patient outcomes. We will use SPSS version 20.0 for data analysis. Significance for all analyses will be set at *P* < 0.05.

### Process evaluation

A process evaluation, according to Hulscher et al. [42], will be performed during and after the intervention to investigate the feasibility of the implementation strategy and to gain insight into the impact of individual implementation activities on guideline adherence. For this purpose, we will document the different implementation activities and the participation level of the health-care providers in order to determine the correlation between the implementation effect (i.e., results of our implementation strategy on guideline adherence) and compliance to the implementation strategy (i.e., degree of implementation). For this, we use the framework of Hulscher et al. for describing the key features of an implementation of change intervention [43]. In this framework, attention is paid to features of the target group, features of the implementers, and the frequency and intensity of intervention activities [43]. Based on this framework, we describe the features of the intervention as performed in detail. The process evaluation will furthermore be based on a questionnaire for the contact persons and a questionnaire for the health-care providers to measure their experience with the implementation strategy. The survey will be emailed to those who have participated in an activity. We will also determine other factors that could have influenced adherence to the perioperative safety guidelines, such as local interventions in the hospitals, audits by the IGZ, other scientific studies in this field conducted in the participating hospitals, and possible confounding factors and barriers for guideline adherence like changing registration systems in the hospitals or merging hospitals.

### Economic evaluation

Better adherence to the perioperative guidelines may reduce unnecessary perioperative harm such as complications and subsequently welfare losses. To assess the cost implications, an economic evaluation will be conducted that is based on the general principles of a cost-effectiveness analysis. The perspective of this economic evaluation will be a health-care perspective. The input of resources in the implementation strategy will be assessed by collecting volumes of consumed resources and multiplying these by the price of each resource unit (market prices, guideline prices or self-determined prices based on costing methods, i.e., full costing) [44]. The implementation process and consequent costs will be estimated by an activity-based costing (ABC) approach focusing on activities performed with costs accumulated at the activity level(s) of the health-care implementation process [44]. ABC attempts to identify all subprocesses related to the implementation process. Next, the underlying activities (personnel, material, and overhead costs) associated with these subprocesses are identified. Use of ABC concepts facilitates the identification of no value-added activities. The output or consequences of the implementation strategy will be determined by the level of adherence to the perioperative guidelines, measured before and after implementation support.

### Ethical approval

This study protocol was approved by the Medical Ethical Committee of the Radboud university medical center on 9 August 2011 (registration number: 2011/318). The study conforms to Dutch law and privacy regulations and was judged not to involve human-subject research. This study is registered in the Dutch Trial Registry for clinical trials [45] (record number NTR3568). The participation of hospitals in the study is voluntary. The research team gave oral presentations and written information about the study to each hospital. Written consent has been obtained from all participating hospitals before randomization. Because outcome data are routinely collected by the hospitals and no personal identifiers are transmitted, individual consent of patients is not required. All data will be handled strictly confidential and published anonymously. Each participant is identified in the database with a study number.

### Trial status

Between May 2012 and June 2015, nine hospitals have been found willing to participate in this study. At the time of submission of the manuscript, no data cleaning or analysis has begun.

## Discussion

From the literature, we know that adherence to guidelines is too low [46] and because of this the safety of patients is at stake. This is not different for the guidelines on the perioperative process. Many potentially effective implementation strategies are available [20], but the understanding of whether and why a strategy is successful is still limited; some implementation strategies have been found to be invariably effective in some settings, but not in others [19]. The perioperative setting is an unexplored area with respect to implementation topics. Our study will provide a better understanding of guideline implementation within the perioperative care process. With this study, we hope to show that a tailored multifaceted implementation strategy will be as successful in the perioperative setting as in other settings [20]. This is important for the further implementation of the perioperative guidelines in all Dutch hospitals. To the best of our knowledge, this is the first study examining the effects of a multifaceted approach in the perioperative care setting. Knowledge on the effects of our multifaceted approach to implement the perioperative safety guidelines can be used to improve perioperative patient safety.

This study has several strengths and limitations. The major strength of this study is the randomized comparison made possible by the multicenter stepped wedge cluster randomized trial design. In fact, a parallel group cluster randomized trial was not feasible because of the large number of hospitals needed. A (individually) randomized, controlled trial was not possible as all Dutch hospitals have a legal obligation to implement the perioperative safety guidelines; temporarily postponing the implementation of the guidelines would have been unethical. Finally, non-randomized designs such as pre- and post-intervention evaluations are methodologically weaker as they tend to overestimate intervention effects. Another strength of our study is the use of a multifaceted approach consisting of in total 11 different activities, covering six interventions: education, audit and feedback, organizational interventions, team-directed interventions, reminders, as well as patient-mediated interventions, of which most have been shown to be effective in other studies [20,41,47-52]. Combinations of interventions may be more effective than single interventions in changing professional behavior, because a larger variety of barriers for change can be addressed [53]. The effectiveness of a combined strategy is determined by the effectiveness of the separate interventions that make up the strategy and the interaction of the different interventions, which can increase or reduce the total effect. The effect of a combined intervention does not necessarily equal the sum of the effects of the single interventions that comprise it. Different interventions can support each other, so the total effect may be larger than the effects of the interventions separately [53]. However, no specific combination provides a guarantee for success [53]. It seems plausible that combined interventions are only more effective than a single intervention if they address barriers and facilitators that are actually related to professional performance. This emphasizes the importance of a careful documentation of the most important barriers to improvement and the systematic development of implementation strategies as in our study, which is preferred over intuitively selected strategies [53,54]. A multifaceted implementation strategy with activities that are not carefully related to the relevant factors and needs of the target group is an unfocused intervention and likely not effective [53].

The generalizability of the findings will be high because the study is carried out in a large, heterogeneous sample of nine hospitals across the country. A further strength of this study is the wide scope; we perform measurements at the cluster level and the level of their individual members, i.e., care providers and patients. A questionnaire is sent to the key disciplines to explore all relevant barriers and facilitators to guideline adherence and opportunities for improvement. A better understanding of the barriers and facilitators and a strategy tailored to local circumstances will enhance the implementation of the perioperative guidelines like already mentioned [55,53]. We also carry out a process evaluation to analyze the mechanisms and processes responsible for the outcomes of the effect evaluation; a causal relationship between implementation activities and change in outcomes is difficult to identify because of the presence of confounding factors, like local hospital initiatives to improve patient safety. In case of a multifaceted implementation strategy, the process evaluation is especially important to specify which intervention was most effective [53]. Insight into the implementation process is also relevant for other hospitals that can benefit from the lessons learned. To improve the internal validity, the following actions will be undertaken. To reduce selection bias, the first 50 patients that meet the inclusion criteria are selected from the hospital databases in all hospitals at each measurement point. A possible limitation of the survey investigations is response bias, which will be minimized by sending reminders. Information bias is reduced by having the same researchers (YE and GB) checking the data from the hospital registries and medical records. An independent researcher checks the data entries. An independent statistician will check the analyses. To prevent confounding bias, we study potentially confounding determinants, such as team climate and patient safety culture. To prevent publication bias, the study has been registered in the Dutch register for clinical trials. Additionally, we have installed an external advisory board with the special tasks of checking the design and the integrity of the study and giving feedback.

With respect to the limitations of the study, the power of the stepped wedge cluster randomized trial design decreases if the treatment gradually reaches its expected effect after one or more time intervals after the intervention [22]. The greater the delay in the intervention effect, the greater the loss of power. This can be a problem in the third group of hospitals as the effect measurement is conducted immediately after the implementation phase (the last group starts with the implementation activities after T2). It is therefore important to make the time intervals sufficiently long so that the full intervention effect is realized in a single interval. The power can also be partly, but not completely, restored by adding additional measurement periods onto the end of the trial. Another limitation of this study is the costs of the frequent data collection. If the necessary data are not easy to collect and time-consuming, the cost of data collection is substantial. A further limitation is due to the fact that our sample size is calculated for the primary outcome measure. Power may be too low to draw conclusions about the effect of guideline adherence on perioperative patient safety. Blinding of the intervention is not possible as the trial is designed to evaluate an implementation intervention to change professional behavior in the real hospital setting. Outcomes assessment was not blind either, but as indicators are rather objective (e.g., is a protocol for anticoagulant use available or not), it is unlikely that they are influenced by the assessment.

Overall, knowledge of the results of this study will improve the implementation success of the perioperative guidelines. It is expected that better adherence to the perioperative safety guidelines will improve the safety of perioperative care.

## Additional file

Additional file 1:
**Supplementary Tables.** This file contains CONSORT 2010 checklist of information to include when reporting a cluster randomized trial and extension of CONSORT for abstracts to reports of cluster randomized trials.
